# *Lodderomyces elongisporus*: An emerging human fungal pathogen

**DOI:** 10.1371/journal.ppat.1011613

**Published:** 2023-09-07

**Authors:** Yue Wang, Jianping Xu

**Affiliations:** Department of Biology, McMaster University, Hamilton, Ontario, Canada; University of Maryland, Baltimore, UNITED STATES

*Lodderomyces elongisporus*, a diploid ascomycete yeast, is attracting broad attention due to its increasing infection of humans. First discovered and described as *Saccharomyces elongisporus* in 1952 from Californian citrus concentrate [1], this yeast has since been isolated from many sources, including soil, fermented food products, plants, stored apples, pigeon excreta, insects, marine fish, hospital environments, and humans [2–6]. Its medical relevance was first noted in 2008 when a retrospective analysis of 542 clinical *Candida parapsilosis* isolates from 25 countries revealed that ten isolates were actually *L*. *elongisporus* [7]. Since then, infections caused by this fungus have been reported in 14 countries on 5 continents (Fig 1; Table 1).

**Fig 1:**
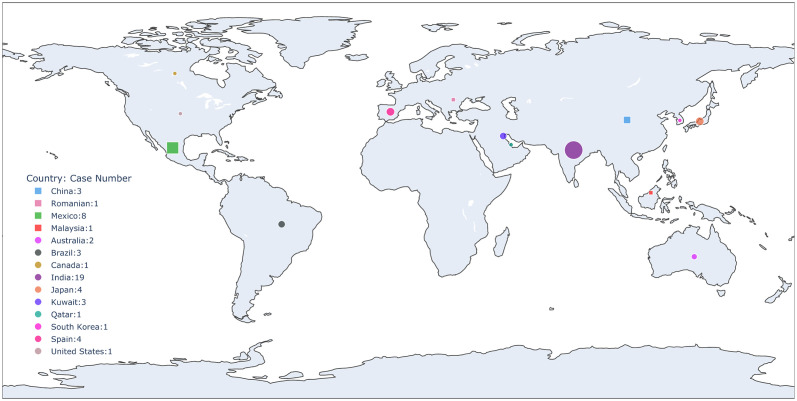
Geographic distribution of *L*. *elongisporus* cases. The figure was generated using python *plotly*. Here, cases from retrospective surveys were marked with squares, while those from published case reports were marked with circles.

**Table 1:** Reported cases of *Lodderomyces elongisporus* infections.

Case	Age/sex	Country	Source	Therapy	Outcome	Underlying diseases
2012 [14]	30 yr/M	Australia	blood, valvular tissue	CAS -> 5-FC, Amp B, VOR	survived	depression, OM, ED, BL, IDU
2013 [9]	63 yr/M	Kuwait	CVC tip	FLU + CVC removal	died	SZA, HD, LH, seizure
2014 [33]	22 yr/M	Qatar	blood	CAS	died	trauma
2016 [24]	39 yr/M	Japan	blood, CVC tip	MICA + CVC removal	survived	aorto-esophageal fistula
2017 [34]	79 yr/M	Spain	blood	CAS	died	COPD, DM, ESRD
2018 [35]	71 yr/F	Kuwait	blood	CAS	died	hypertension, HD, PVD
2018 [26]	56 yr/F	South Korea	blood, PICC	NA	died	cancer
2020 [36]	54 yr/M	Australia	blood	ANI + line removal	survived	pseudo-obstruction, SBS
2021 [17]	62 yr/M	Canada	arachnoid biopsy	Amp B + VOR -> FLU	survived	cancer, lymphopenia, meningitis
2021 [15]	46 yr/M	USA	blood	MICA->Amp B + 5-FC	survived	ED, ICH, HD, IDU
2022 [19]	65 yr/F	Spain	vaginal	FLU	survived	NA
	22 yr/F		endocervical exudate	CLOT		Chlamydia trachomatis infection
	adult/F		vaginal	CLOT		NA
2022 [37]	9 day/F	Kuwait	blood	Amp B	died	LBW, HMD
2023 [38]	infant/M	India	blood	FLU (*n* = 3)	survived (*n* = 6), LAMA (*n* = 1), NA (*n* = 1)	sepsis
	infant/M					HD, kidney atrophy (neonate)
	infant/M					TEF (4 months)
	adult/F					kidney injury (*n* = 2), MAT (*n* = 1), DM (*n* = 1), cancer (*n* = 1)
	adult/F					
	adult/M					
	adult/M					
	adult/M					
2023 [39]	25 wk (GA)/F	Brazil	blood	NA	died	PT, LBW
2023 [18]	adult/NA	Brazil	oral mucosa	NA	NA	AIDS
	adult/NA					
2023 [23]	76 yr/M	Japan	blood, CVC tip	MICA + CVC removal	survived	DM, aneurysm, angina
	12 yr/M		blood, CVC tip	MICA + CVC removal	survived	SAH, autism
	82 yr/F		blood	fosFLU -> CAS	survived	DM, BL, PD, cholangitis
2023 [16]	11 yr/M	India	blood	Amp B	LAMA	VSD, HD, ED
2023 [6]	10 day/NA	India	blood	FLU + Amp B	survived	PT, LBW, TP
	23 day/NA		blood		survived	PT, IUGR, TP
	11 day/NA		blood		survived	PT, LBW, asphyxia
	14 day/NA		blood		survived	PT, LBW, TP, hypoglycemia
	19 day/NA		blood		survived	hypoglycemia, TP
	16 day/NA		blood		survived	PT, LBW, TP
	30 day/NA		blood		died	PT, LBW
	7 day/NA		blood		survived	LBW, sepsis, TP
	7 day/NA		blood		survived	LBW, asphyxia
	10 day/NA		blood		survived	PT, LBW

LBW, low birth weight; TP, thrombocytopenia; DM, diabetes mellitus; HD, heart disease; PT, preterm; ED, endocarditis; VSD, ventricular septal defect; SAH, subarachnoid hemorrhage; IUGR, intrauterine growth restriction; PVD, peripheral vascular disease; ICH, intracerebral hemorrhage; PD, Parkinson’s disease; MAT, mesenteric artery thrombosis; TEF, tracheoesophageal fistula; HMD, hyaline membrane disease; SBS, short bowel syndrome; BL, brain lesions; OM, osteomyelitis; SZA, schizoaffective disorder; LH, left hemiplegia; COPD, chronic obstructive pulmonary disease; ESRD, end-stage renal disease; AIDS, acquired immunodeficiency syndrome; IDU, intravenous drug user; CAS, caspofungin; 5-FC, flucytosine; Amp B, amphotericin B; FLU, fluconazole; VOR, voriconazole; MICA, micafungin; ANI, anidulafungin; CLOT, clotrimazole; fosFLU, fosfluconazole; PV, peripheral venous catheter; CVC, central venous catheter; PICC, peripherally inserted central catheter; LAMA, left against medical advice; GA, gestational age; yr, year; wk, week; F, female; M, male; NA, not available.

In 1966, the genus name *Lodderomyces* was proposed as a replacement for the original name *Saccharomyces elongisporus* to highlight its different physiological features from the type species of *Saccharomyces* [8]. Despite the unique genus name, *L*. *elongisporus* closely resembles *C*. *parapsilosis*: they both grow as oval to elongated cells or in pseudohyphal form, forming cream-colored colonies on Sabouraud dextrose agar. Furthermore, they both can assimilate high molecular-weight paraffins. Therefore, clinical isolates of *L*. *elongisporus* have often been misidentified as *C*. *parapsilosis* by conventional methods such as API 20C, ID 32C, and Vitek 2 [7,9]. However, researchers noticed this mistake after plating isolates previously identified as *C*. *parapsilosis* isolates on CHROMagar and finding that certain isolates formed turquoise blue colonies rather than the white to pale pink colonies typical of *C*. *parapsilosis*. Subsequent molecular analysis revealed that isolates forming turquoise blue colonies actually belonged to *L*. *elongisporus*.

Phylogenetic analyses based on DNA sequences at a single gene, multiple genes, and whole genomes all clustered *L*. *elongisporus* into the *Candida* clade, closely related to the *C*. *parapsilosis* species complex that includes *C*. *parapsilosis*, *Candida orthopsilosis*, and *Candida metapsilosis* [10] (Fig 2). The *L*. *elongisporus* genome size (15 to 16 Mb) is slightly larger than that of *C*. *parapsilosis* (12 to 13 Mb) but in the range of several other common human pathogenic *Candida* species such as *Candida albicans* (14 to 16 Mb) and *Candida tropicalis* (14 to 15 Mb). Species in this clade also shared comparable gene numbers and a largely conserved gene order [11]. Importantly, similar to other species in this clade, the CUG codon in *L*. *elongisporus* is translated to serine instead of leucine as in most other organisms [11]. Together, these characteristics suggest that *L*. *elongisporus* should probably be changed to *Candida elongisporus*.

**Fig 2:**
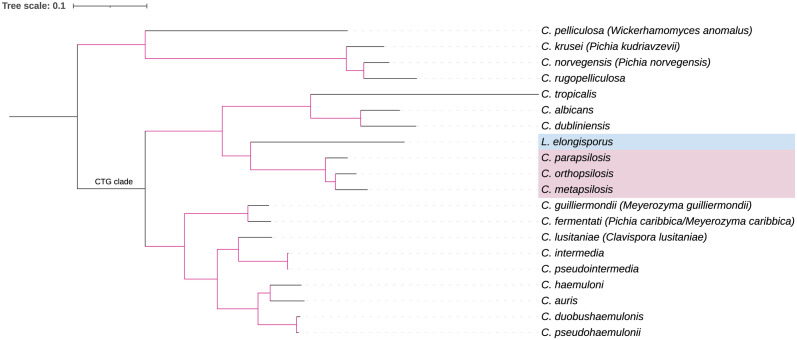
Maximum-likelihood tree showing phylogenetic relationships between *L*. *elongisporus* and representative species in the genus *Candida*. The tree was constructed based on concatenated DNA sequences of conserved mitochondrial protein-coding genes using *FastTree*. Species names in parenthesis are recently updated names for those organisms. The purple branches represent those with over 94% bootstrap support, based on 1,000 resamples.

## Unique features of *L*. *elongisporus* as compared to other species within the CTG clade

*Candida* species that translate the CUG codons as serine rather than leucine belong to the commonly called CTG clade [12]. This clade is notable because most of its members can cause diseases in humans. However, species within the CTG clade can vary significantly in several features, including their relative prevalence in humans, stress tolerance, and virulence properties [13]. For example, *C*. *albicans*, *C*. *tropicalis*, and *C*. *parapsilosis* are common agents of fungemia in humans while *L*. *elongisporus* and several others are infrequent pathogens. Second, compared to *C*. *albicans* and *C*. *tropicalis*, *L*. *elongisporus* is generally more sensitive to stressors such as salinity, H_2_O_2_, and pH fluctuation. Third, when phagocytosed, *C*. *albicans* and *Candida dubliniensis* often exhibit filamentous forms, while *L*. *elongisporus* remains in the yeast form. Fourth, both *L*. *elongisporus* and its close relative *C*. *parapsilosis* produce less biofilm than *C*. *albicans*, *C*. *tropicalis*, and *C*. *dubliniensis* on polystyrene surface [13].

Multiple species in the CTG clade, including *C*. *albicans*, *Candida guilliermondii*, *Candida famata*, and *Candida lusitaniae*, are capable of mating and/or undergoing sexual reproduction. Although *L*. *elongisporus* was previously suggested to be homothallic [8], its sexual reproductive structure, including multiple ascospores within each ascus, was not described until recently. Similarly, early comparative genomic analysis revealed that all 4 mating-type genes (MTLa1p, MTLa2p, MTLα1p, and MTLα2p) found in most other species in the CTG clade were apparently missing in *L*. *elongisporus* [11]. However, recent research on strains from India identified that all 19 Indian *L*. *elongisporus* isolates had sequences that partially match to the MTLa1p of *C*. *parapsilosis* and MTLα2p of *C*. *albicans*, while no match was found in *L*. *elongisporus* genomes to either MTLa2p or MTLα1p [6]. Surprisingly, despite not containing the complete mating loci, all bloodstream isolates and several environmental isolates from India developed asci containing multiple ascospores on acetate ascospore agar, suggesting *L*. *elongisporus* is capable of sexual reproduction [6]. In addition, loss of heterozygosity and signatures of recombination (including phylogenetic incompatibility and linkage equilibrium) were observed in the Indian population of this diploid yeast, consistent with secondary homothallism and/or frequent mitotic recombination for this species in the environment and in clinics [6].

## Risk factors for *L*. *elongisporus* infections and treatment

To date, 39 cases with *L*. *elongisporus* infections have been reported (Table 1), among which 17 patients were males, 10 females, and 12 not specified. Among the 39 cases, 34 were systemic, including 33 bloodstream infections (of which 3 also had endocarditis) [14–16], and 1 meningitis [17]. Of the remaining five, 2 were oropharyngeal candidiasis and 3 were vaginitis [18,19]. Among the diseased patients, 9 were 50 years or older, 7 were between 10 and 49 years, 14 were neonates, and 9 unspecified, suggesting both elderly individuals and neonates are at risk of developing *L*. *elongisporus* infections. In addition, most patients with invasive infections had underlying conditions. For example, 4 of 7 patients over 60 years old had diabetes, and of the 14 neonates, 10 were born with low birth weight, 8 were premature, and 6 had thrombocytopenia (Table 1). Prior to fungal infections, 10 patients had catheters and 14 received antibiotic therapy.

Over the years, there has been a rising frequency of invasive candidiasis and along with it, an increasing mortality. Estimated crude in-hospital mortality related to invasive candidiasis is around 25% but the mortality rate differs among patient populations [20]. Among the reported invasive infections by *L*. *elongisporus*, 8 of 34 patients died, a rate (23.5%) similar to the overall mortality reported for invasive candidiasis. However, since most patients that developed invasive *L*. *elongisporus* infections also suffered from other comorbidities, the relative contribution of invasive *L*. *elongisporus* infection to the death of individual patients is often not clear.

So far, clinical isolates of *L*. *elongisporus* have shown an overall low level of minimal inhibitor concentrations (MICs) to most antifungal drugs. The reported MIC (μg/mL) ranges for clinical *L*. *elongisporus* isolates are: flucytosine: 0.06–1; amphotericin B: 0.012–0.5; fluconazole: 0.12–1; itraconazole: 0.008–0.25; voriconazole: 0.0017–0.12; posaconazole: 0.003–0.5; micafungin: 0.003–1; caspofungin: 0.008–0.5; and anidulafungin: 0.015–0.5. These MIC values are lower than most of those reported for *C*. *albicans*, *C*. *tropicalis*, and *C*. *parapsilosis* [21]. For example, despite the close evolutionary relationship between *L*. *elongisporus* and *C*. *parapsilosis*, the MICs of *L*. *elongisporus* to echinocandins are closer to *C*. *albicans* than to *C*. *parapsilosis*. This was largely due to the unique amino acid sequence of beta-1,3 glucan synthase, the target of echinocandins, in *C*. *parapsilosis* [22]. Indeed, echinocandins are highly effective for treating *L*. *elongisporus* infections but not for *C*. *parapsilosis* infections. Thus, it’s important to distinguish between *L*. *elongisporus* and *C*. *parapsilosis* isolates (as well as other pathogenic yeasts) in clinical microbiology labs and failure to do so can have significant treatment implications. However, 2- to 4-fold elevated fluconazole MICs have been observed in several environmental isolates [6], indicating that *L*. *elongisporus* can evolve resistance to antifungal drugs. Together, these results suggest continued monitoring of antifungal susceptibility patterns is required in order to develop appropriate treatment strategies.

## Potential transmission routes of *L*. *elongisporus*

*Lodderomyces elongisporus* can survive in the hospital environment. Reports have shown patients developing *L*. *elongisporus* infections while receiving treatments for other comorbidities during their stay in hospitals [6,23,24], suggesting these infections were likely hospital-acquired. The ability to form ascospores and biofilms likely contributes to *L*. *elongisporus* survival in the clinical inanimate environment. Compared to vegetative cells, ascospores are usually more resistant to environmental pH fluctuation and extreme temperatures [25]. While phenotypic analysis based on a single strain revealed a limited ability of biofilm formation in *L*. *elongisporus*, the fact that strains were isolated from the catheter tip of patients suggests that biofilm formation ability is present in at least some clinical strains in vivo [9,23,24,26]. Moreover, elevated MICs against sodium hypochlorite, a common disinfecting agent, have been observed in multiple clinical-related isolates [6]. Together, these observations suggest that *L*. *elongisporus* possesses diverse features to enable its persistence in clinical settings.

Indeed, transmission of this species has been observed within hospital environments. For example, 10 neonates in the span of 6 months developed fungemia caused by *L*. *elongisporus*. These blood culture isolates were genetically very similar to each other [6]. Further investigation of the hospital environment revealed that the railing and the temperature panel of the open care warmer used by the infected neonates were also colonized by *L*. *elongisporus*. Genomic analysis confirmed the close relatedness of these hospital environmental strains and neonate strains. Interestingly, this study also reported that several *L*. *elongisporus* isolates from fruit surfaces were genetically very similar to the clinical isolates. Taken together, the results suggested that this yeast can be transmitted within and outside hospitals.

Aside from humans, *L*. *elongisporus* can also infect wild and domesticated animals. One report described a dog being quilled by a porcupine and subsequently developed pericarditis and endocarditis due to *L*. *elongisporus* infection [27]. Another study reported a porcupine with alopecia and dermatitis caused by *L*. *elongisporus* [28]. These studies suggest that porcupines could be a potential reservoir and/or carrier of *L*. *elongisporus*. The significance of animal and other sources of *L*. *elongisporus* in relation to human infections remains to be determined.

## In-depth investigations of *L*. *elongisporus* are needed

Our literature search revealed that globally, 18 case studies have reported *L*. *elongisporus* infections. In addition, several retrospective analyses revealed that many clinical “*Candida parapsilosis*” isolates actually belonged to *L*. *elongisporus*. For example, 1 retrospective survey conducted in 2008 revealed that 10 of the 542 “*Candida parapsilosis*” isolates from blood samples from Mexico, Malaysia, and China were *L*. *elongisporus* [7]. In 2014, a study analyzing 389 “*Candida*” isolates from 244 patients in multiple intensive care units in China identified 2 *L*. *elongisporus* strains that caused invasive candidiasis [29]. In 2015, a Romanian multicenter study discovered 1 strain of this fungus from 551 clinical yeast isolates [30].

*Candida parapsilosis* is the second or third most frequent cause of candidemia in many geographic regions. Due to its high similarity to *C*. *parapsilosis*, many previously identified *C*. *parapsilosis* infections were likely caused by *L*. *elongisporus*. Thus, the prevalence of *L*. *elongisporus* infections is likely much higher than currently reported [31]. Accurate identification techniques, e.g., ITS sequencing and MALDI-TOF MS, are recommended to reveal the true incidence of *L*. *elongisporus* and other rare fungal pathogens. Broad ecological sampling and genotyping is needed to understand its environmental reservoirs, relationships among ecological samples, and potential threats ecological niche populations to humans and other animals [32]. Furthermore, its ability to adapt to growing at high concentrations of chemical disinfectants [6] calls for greater efforts to understand its genetic mechanisms of stress tolerance and how best to eliminate *L*. *elongisporus* from hospital environments.
